# Gypenoside attenuates hepatic ischemia/reperfusion injury in mice via anti-oxidative and anti-apoptotic bioactivities

**DOI:** 10.3892/etm.2014.1569

**Published:** 2014-02-21

**Authors:** JIE ZHAO, YINGZI MING, QIQUAN WAN, SHAOJUN YE, SONG XIE, YI ZHU, YANFENG WANG, ZIBIAO ZHONG, LING LI, QIFA YE

**Affiliations:** 1 Center of Transplant Medicine Engineering and Technology of the Ministry of Health of The People’s Republic of China, The Third Xiangya Hospital, Central South University, Changsha, Hunan 410013, P.R. China; 2 Institute of Hepatobiliary Disease, Transplant Center, Zhongnan Hospital, Wuhan University, Wuhan, Hubei 430071, P.R. China

**Keywords:** ischemia/reperfusion, hepatic injury, gypenoside, oxidative damage, apoptosis

## Abstract

*Gynostemma pentaphyllum* is a traditional Chinese medicine that has previously been used for the treatment of chronic inflammation, hyperlipidemia and liver disease. Gypenoside (GP), the predominant component of *Gynostemma pentaphyllum*, exhibits a therapeutic effect on chronic hepatic injury, fibrosis and fatty liver disease via its anti-inflammatory and anti-oxidant activity. However, the effect of GP on ischemia/reperfusion (I/R)-induced hepatic injury has, to the best of our knowledge, not previously been investigated. In the present study, a hepatic I/R-injury model was successfully established using C57BL/6 mice. In the treatment group, 50 mg/kg GP was administered orally 1 h prior to ischemia. Following hepatic I/R, the levels of hepatic lipid peroxidation and serum alanine aminotransferase increased, while the ratio of hepatic glutathione (GSH):oxidized GSH was reduced, which was effectively attenuated by pretreatment with GP. Furthermore, an increased protein expression of heme oxygenase-1 in the liver tissues of the I/R mice was attenuated by the administration of GP. In addition, the present study indicated that treatment with GP suppressed the I/R-induced increase in the pro-apoptotic protein levels of Bax and cytochrome *c* and the activity of caspase-3/8, as well as the I/R-induced decrease in the levels of anti-apoptotic protein Bcl-2. In conclusion, the present study indicated that GP effectively protected against I/R-induced hepatic injury via its anti-oxidative and anti-apoptotic bioactivity.

## Introduction

Ischemia/reperfusion (I/R) is a predominant cause of hepatic injury, which is of clinical significance following liver surgery, hemorrhagic shock and liver transplantation (1). Therefore, developing effective preventive and therapeutic strategies for I/R-induced hepatic injury is required.

It has been demonstrated that I/R-induced hepatic injury is initially triggered by reactive oxygen species (ROS), which induce oxidative damage and apoptosis, an important mechanism for cell death following hepatic I/R injury (2). In addition, certain pro-inflammatory chemokines and cytokines are key during the initial period of reperfusion, whereas the late period of hepatic injury is neutrophil-mediated (3).

*Gynostemma pentaphyllum* is a traditional Chinese medicine, which has previously been used for the treatment of chronic inflammation, hyperlipidemia and liver diseases (4). Gypenoside (GP) is the predominant component of *Gynostemma pentaphyllum* and has been shown to possess anti-inflammatory, antitumor and anti-oxidant capabilities (5–7). Furthermore, GP exhibits a therapeutic effect on chronic hepatic injury, fibrosis, as well as fatty liver disease, which were induced by a high fat, high cholesterol diet and alcohol in mice (5,8). Qi *et al* (9) reported that GP protected against DNA damage in neurons in I/R-induced cerebral injury. To the best of our knowledge, the role of GP in hepatic I/R injury and the underlying molecular mechanism, have not yet been investigated.

Therefore, in the present study the protective effects of GP against I/R-induced hepatic injury in mice was studied. In addition, the molecular mechanisms involved were investigated, particularly regarding oxidative stress and apoptosis.

## Materials and methods

### Hepatic I/R procedure

All of the methods in the present study were approved by the Ethics Committee of Xiangya Hospital of Central South University (Changsha, China) and were performed according to the Guidelines for Care and Use of Experimental Animals published by the Chinese Association for Laboratory Animal Sciences. Male C57BL/6 mice (age, 8–12 weeks; weight, 20–25 g) were housed in a laminar flow, temperature-controlled, pathogen-free environment under a 12 h light/dark cycle, at Xiangya Medical Experimental Animal Center of Central South University (Changsha, China).

Prior to the experiments, the mice were fasted for 24 h and provided with tap water *ad libitum*. During the procedure, xylazine (7 mg/kg body weight) and ketamine (55 mg/kg body weight) were administered as an anesthetic. To induce ischemia of the median and left lobes of the liver, a micro clip was used to clamp the left branches of the portal vein and hepatic artery following a transverse incision, which was made to the abdomen. The clamp was removed after 1 h and the wound was closed. Six hours following reperfusion, the mice were sacrificed by cervical dislocation under anesthesia, and the ischemic liver tissues and blood were collected immediately. The same procedure was performed in the sham group but without vessel occlusion.

### Administration of GP

GP was purchased from the China National Pharmaceutical Group Corporation (Beijing, China) and dissolved in saline according to the manufacturer’s instructions. Thirty minutes prior to ischemia, 50 mg/kg GP was administered orally. In the present study, the mice were divided into four groups as follows: i) Vehicle-treated sham; ii) GP-treated sham; iii) vehicle-treated I/R; and iv) GP-treated I/R. As there were no differences identified in any of the parameters between the vehicle-treated and GP-treated sham groups, these two groups were pooled and referred to as the sham group.

### Hepatic lipid peroxidation and glutathione (GSH) content

The level of thiobarbituric acid reactive substances in the liver homogenates was measured spectrophotometrically (Shimadzu, Kyoto, Japan) at a wavelength of 535 nm to determine the steady-state level of malondialdehyde (MDA), which is the end-product of lipid peroxidation. 1,1,3,3-tetraethoxypropane (Sigma-Aldrich, St. Louis, MO, USA) served as the standard. Following precipitation with 1% picric acid, the GSH level was determined in the liver homogenates using yeast-GSH reductase, 5,5′-dithio-bis (2-nitrobenzoic acid) and nicotinamide adenine dinucleotide phosphate, at a wavelength of 412 nm. The same method was used to measure the oxidized GSH (GSSG) level in the presence of 2-vinylpyridine; the GSH:GSSG ratio was subsequently calculated.

### Serum aminotransferase activity

The blood was collected and alanine aminotransferase (ALT) and aspartate aminotransferase (AST) assay kits (Biovision, Milpitas, CA, USA) were used to determine the activity of serum ALT and AST, respectively, in accordance with the manufacturer’s instructions.

### Activity of caspase-3 and -8

In accordance with the manufacturer’s instruction, caspase-3 and-8 colorimetric assay kits (Biovision, San Francisco Bay Area, CA, USA) were used to determine the activity of caspase-3 and -8, respectively. Briefly, 20 μg liver cytosolic protein was extracted and incubated in a solution buffer at room temperature for 30 min. The caspase reaction was initiated by adding 200 μM N-acetyl-Asp-Glu-Val-Asp-7-amino-4-trifluoromethylcoumarine and incubated at 37°C for 2 h. The change in fluorescence (excitation at 400 nm) was detected.

### Western blot analysis

Cytoplasmic extraction reagents (Pierce Biotechnology, Rockford, IL, USA) were used to obtain cytosolic proteins from the mice liver tissues, in accordance with the manufacturer’s instructions. Following the determination of the concentration of protein using the protein assay reagents, 20 μg protein was separated using 10% SDS-PAGE and transferred to nitrocellulose membranes, which was maintained at room temperature for 1 h in a buffer solution containing 5% dried skimmed milk. The membrane was incubated at room temperature for 3 h with mouse anti-heme oxygenase-1 (HO-1) monoclonal antibody (1:400; Abcam, Cambridge, UK), mouse anti-cytochrome *c* monoclonal antibody (1:200; Abcam), mouse anti-Bcl-2 monoclonal antibody (1:200; Abcam), mouse anti-Bax monoclonal antibody (1:200; Abcam) or mouse anti-GAPDH monoclonal antibody (1:200; Abcam), respectively, and with rabbit anti-mouse secondary antibody conjugated to horseradish peroxidase (1:10,000; Abcam) for 1 h. The signals on the membranes were detected using an enhanced chemiluminescence reagent (PerkinElmer, Waltham, MA, USA) and the densitometry was analyzed using Image-Pro plus software 6.0 (Media Cybernetics, Inc., Rockville, MD, USA).

### Statistical analysis

All data are indicated as the mean ± standard error of the mean and were analyzed by one-way analysis of variance. P<0.05 was considered to indicate a statistically significant difference.

## Results

### GP treatment attenuates the increase in hepatic lipid peroxidation and GSH content in hepatic tissue of an I/R mouse model

**Figure 1:**
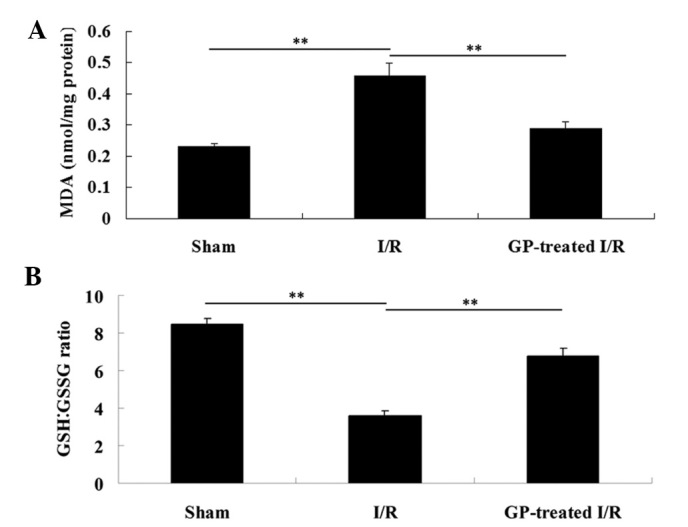
(A) The MDA level was determined in the liver homogenates obtained from each group. (B) The GSH levels and GSSG were determined in the liver homogenates obtained from each group. The GSH:GSSG ratio was calculated. ^**^P<0.01. MDA, malondialdehyde; I/R, ischemia/reperfusion; GP, gypenoside; GSH, glutathione; GSSG, oxidized GSH.

To evaluate the degree of hepatic injury in each group, the hepatic lipid peroxidation and GSH content were investigated in the liver tissue. As shown in Fig. 1A, the MDA level in the sham-operated liver tissues was 0.23±0.01 nmol/mg. However, the MDA level in the I/R group significantly increased to 0.46±0.04 nmol/mg, compared with that in the sham group (P<0.01). In the GP-treated I/R group, the MDA level was significantly reduced compared with the I/R group (P<0.01). In addition, as shown in Fig. 1B, the GSH:GSSG ratio in the I/R group was markedly decreased (from 8.44 to 3.61), when compared with that observed in the sham group (P<0.01), which was attenuated by pre-treatment with GP (P<0.01).

### GP treatment attenuates the increase in activity of serum aminotransferases in the hepatic tissue of an I/R mouse model

**Figure 2:**
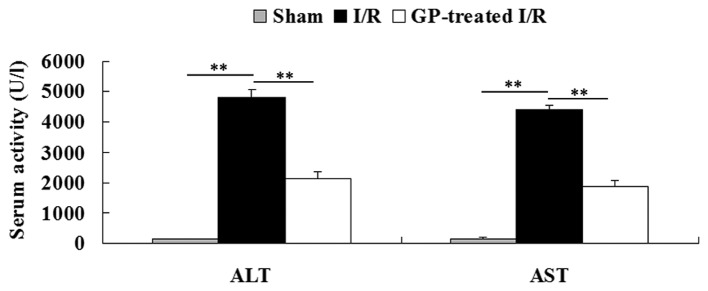
ALT and AST assay kits were used to determine the activities of serum ALT and AST in the blood. ^**^P<0.01. I/R, ischemia/reperfusion; GP, gypenoside; ALT, alanine aminotransferase; AST, aspartate aminotransferase.

The activity of serum ALT and AST was determined in each group. As shown in Fig. 2, the activity of serum ALT and AST was 97.4±9.9 U/l and 107.9±12.1 U/l, respectively, in the sham group. However, the activity of serum ALT and AST in the I/R group was significantly increased (4,833±242 U/l and 4,426±142 U/l, respectively) when compared with those in the sham group (P<0.01). Furthermore, these increases were significantly attenuated by pre-treatment with GP prior to I/R (P<0.01).

### GP treatment attenuates the upregulation of HO-1 protein expression in the hepatic tissue of an I/R mouse model

**Figure 3:**
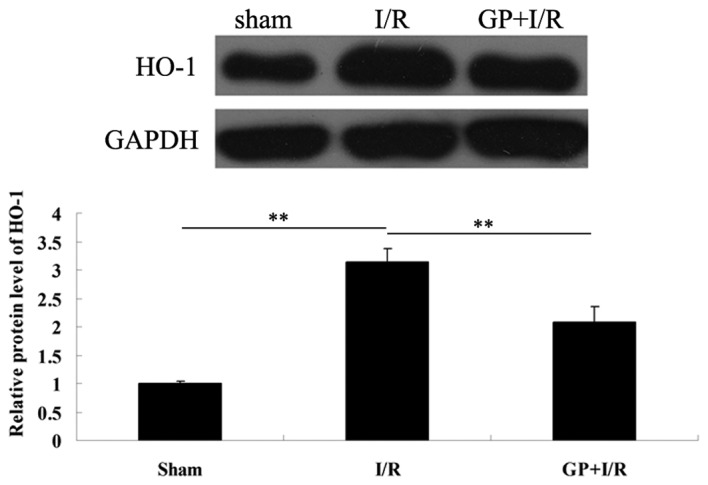
Western blot analysis was performed to determine the protein level of HO-1 in each group. GAPDH served as an internal control. ^**^P<0.01. I/R, ischemia/reperfusion; GP, gypenoside; HO-1, heme oxygenase.

HO-1, a protein that is ubiquitously expressed in various cells, may be markedly induced by numerous stress stimuli, including I/R (10). Therefore, the protein expression of HO-1 was determined in each group. As demonstrated in Fig. 3, the protein expression level of HO-1 notably increased following 6 h of reperfusion compared with the sham group (P<0.01); however, the increase was attenuated by pre-treatment with GP prior to I/R (P<0.01).

### GP treatment attenuates I/R-induced hepatic cell apoptosis by modulating key apoptosis-related proteins

**Figure 4:**
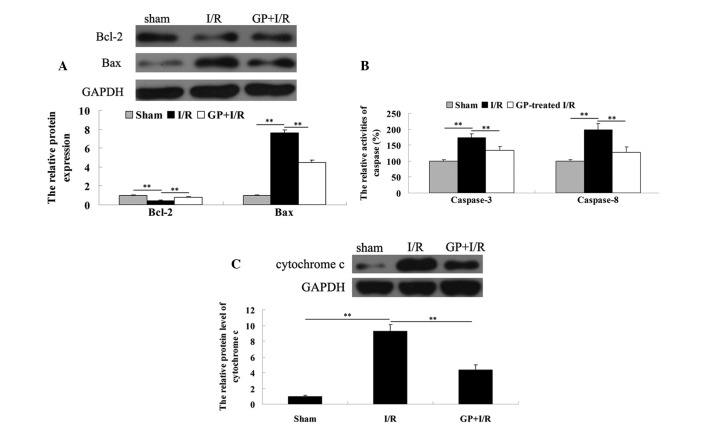
(A) Western blot analysis was performed to determine the protein levels of Bcl-2 and Bax in each group. GAPDH served as an internal control. (B) Caspase-3 and caspase-8 colorimetric assay kits were used to determine the activities of caspase-3 and -8 in each group. (C) Western blot analysis was used to determine the protein levels of cytochrome *c* in the cytosol in each group. GAPDH served as an internal control. ^**^P<0.01. I/R, ischemia/reperfusion; GP, gypenoside.

To determine the apoptosis level in the liver tissues in each group, the expression levels of two apoptotic-related proteins, Bcl-2 and Bax, were determined via a western blot assay. As demonstrated in Fig. 4A, the protein expression level of anti-apoptotic Bcl-2 was lower in the I/R group compared with the sham group, which was attenuated by pre-treatment with GP. Consistently, the protein level of pro-apoptotic Bax was upregulated in the I/R group, when compared with that observed in the sham group, which was markedly attenuated by the administration of GP.

In addition, the activity of caspase-3 and -8, two important indicators for cell apoptosis, was determined. As shown in Fig. 4B, the caspase-3 and -8 activities in the I/R group was significantly increased when compared with that observed in the sham group; however, this increase was attenuated by pre-treatment with GP.

In the I/R group, the protein level of cytochrome *c* in the cytosol was significantly higher following 6 h of reperfusion, when compared with that observed in the sham group. However, these increases were significantly attenuated by pre-treatment with GP (Fig. 4C).

## Discussion

The bioactivity of GP, including anti-inflammatory, anti-oxidative and anti-tumor activity, has been investigated in numerous studies (6,11,12). However, its anti-oxidative capacity is the significant function. It has been demonstrated that reactive oxygen species (ROS) are important in I/R injury, predominantly as ROS initiate lipid peroxidation, leading to structural and functional damage to the organelles (13–15). Furthermore, as endogenous antioxidant levels decrease significantly during I/R, exogenous antioxidants may decrease the severity of I/R damage.

GP has been shown to have a protective role in liver fibrosis, chronic hepatic injury and fatty liver disease (5,16). However, whether GP protects against I/R-induced hepatic injury has not previously been investigated. In the present study, it was hypothesized that GP may attenuate hepatic I/R injury via its anti-oxidative and anti-apoptotic functions.

Reduced GSH has been demonstrated to act as a free radical scavenger and counteract the deleterious effect of ROS (17). In the present study, the administration of GP was shown to effectively attenuate I/R-induced increases in the GSH:GSSG ratio, indicating that GP acts against ROS via reducing GSH during I/R. Furthermore, treatment with GP suppressed the I/R-induced increase in lipid peroxidation. In addition, as HO-1 exhibits a crucial role in the defense against oxidative damage, and overexpression of HO-1 may protect against hepatic I/R injury by modulating oxidative stress (18), the protein level of HO-1 was examined in each group. It was found that GP treatment upregulated the protein expression of HO-1 following I/R. Furthermore, the activity of aminotransferase is an important indicator of oxidative damage (19) and the upregulation of the activity of aminotransferase following I/R was demonstrated to be attenuated by the administration of GP in mice. Therefore, it was hypothesized that GP effectively protects against I/R-induced hepatic injury via suppression of oxidative damage.

Previous studies have indicated that ROS are key in the regulation of cell apoptosis and abundant ROS, resulting from I/R, induce cell apoptosis (2,20). Numerous key factors have been demonstrated to be involved in ROS-mediated cell apoptosis, including the Bcl-2 family, caspase-3/8 and cytochrome *c*. Bcl-2, an anti-apoptotic protein member of the Bcl-2 family inhibits cell endoplasmic reticulum Ca^2+^ release, lipid peroxide formation and free radical production (21). By contrast, Bax, another member of Bcl-2 family, promotes cell apoptosis via a mitochondrial-mediated apoptosis pathway (22). Furthermore, Bax is an endogenous antagonist of Bcl-2, as Bax is able to bind to Bcl-2 via an associated protein that is homologous to Bcl-2, which further inhibits the function of Bcl-2 (23). Notably, Bcl-2 and Bcl-xL form a heterodimer, which is able to suppress the pro-apoptotic effect of Bax (24). In physiological conditions, the protein levels of Bcl-2 and Bax are maintained in equilibrium (22). In the present study, it was hypothesized that GP inhibited I/R-induced cell apoptosis in the liver via a Bcl-2 family-dependent mechanism.

As key members of the cysteine-aspartate-specific protease family, caspase-3 and -8 are ubiquitously expressed in various mammalian cells, and have been demonstrated to be critical in cell apoptosis (25). The activation of caspase-3 and -8 has been observed in various apoptotic cells (26) and upregulation of caspases-3 and -8 has been identified in I/R-induced hepatic injury, indicating that caspase-mediated cell apoptosis is crucial in I/R-induced organ damage (27). Furthermore, an interaction mechanism exists between caspase-3 and Bcl-2; the expression of caspase-3 is negatively regulated by Bcl-2, caspase-3 subsequently hydrolyzes Bcl-2 and the hydrolyzed fragment of Bcl-2 exerts a pro-apoptotic effect (28,29). In the present study, the expression levels of caspase-3 and -8 were investigated and it was identified that GP significantly inhibited the upregulation of these two caspase family members in the I/R-liver tissues in mice. These findings indicated that GP inhibits I/R-induced hepatic cell apoptosis, potentially via the downregulation of caspase-3 and -8. In addition, GP may maintain the balance between caspase-3 and Bcl-2.

Furthermore, during mitochondria-mediated apoptosis, the mitochondrial membrane potential collapses, which leads to further mitochondrial depolarization and cytochrome *c* moves into the cytosol, thus activating an apoptotic cascade (30). Therefore, the cytochrome *c* level in the cytosol was examined in the present study and it was identified that the administration of GP attenuated the increase of cytochrome *c* in the cytosol within I/R-liver tissues.

In conclusion, the present study showed that oral administration of GP effectively protected against I/R-induced hepatic injury, predominantly via the attenuation of oxidative damage as well as by inhibiting cell apoptosis. Therefore, GP may serve as a promising agent for the treatment of hepatic I/R injury.

## References

[1] Mendes-Braz M, Elias-Miró M, Jiménez-Castro MB, Casillas-Ramírez A, Ramalho FS, Peralta C (2012). The current state of knowledge of hepatic ischemia-reperfusion injury based on its study in experimental models. J Biomed Biotechnol.

[2] Yu HC, Bai L, Yue SQ (2013). Notch signal protects non-parenchymal cells from ischemia/reperfusion injury in vitro by repressing ROS. Ann Hepatol.

[3] Zhang A, Chi X, Luo G (2013). Mast cell stabilization alleviates acute lung injury after orthotopic autologous liver transplantation in rats by downregulating inflammation. PLoS One.

[4] Niu Y, Yan W, Lv J, Yao W, Yu LL (2013). Characterization of a novel polysaccharide from tetraploid *Gynostemma pentaphyllum* makino. J Agric Food Chem.

[5] Qin R, Zhang J, Li C (2012). Protective effects of gypenosides against fatty liver disease induced by high fat and cholesterol diet and alcohol in rats. Arch Pharm Res.

[6] Zhang G, Zhao Z, Gao L (2011). Gypenoside attenuates white matter lesions induced by chronic cerebral hypoperfusion in rats. Pharmacol Biochem Behav.

[7] Zhang GL, Deng JP, Wang BH (2011). Gypenosides improve cognitive impairment induced by chronic cerebral hypoperfusion in rats by suppressing oxidative stress and astrocytic activation. Behav Pharmacol.

[8] Chen JC, Tsai CC, Chen LD, Chen HH, Wang WC (2000). Therapeutic effect of gypenoside on chronic liver injury and fibrosis induced by CCl4 in rats. Am J Chin Med.

[9] Qi G, Zhang L, Xie WL, Chen XY, Li JS (2000). Protective effect of gypenosides on DNA and RNA of rat neurons in cerebral ischemia-reperfusion injury. Acta Pharmacol Sin.

[10] Zhang SC, Shi Q, Feng YN, Fang J (2013). Tissue-protective effect of glutamine on hepatic ischemia-reperfusion injury via induction of heme oxygenase-1. Pharmacology.

[11] Aktan F, Henness S, Roufogalis BD, Ammit AJ (2003). Gypenosides derived from *Gynostemma pentaphyllum* suppress NO synthesis in murine macrophages by inhibiting iNOS enzymatic activity and attenuating NF-kappaB-mediated iNOS protein expression. Nitric Oxide.

[12] Chen JC, Chung JG, Chen LD (1999). Gypenoside induces apoptosis in human Hep3B and HA22T tumour cells. Cytobios.

[13] Farmer EE, Mueller MJ (2013). ROS-mediated lipid peroxidation and RES-activated signaling. Annu Rev Plant Biol.

[14] Shi Y, Pulliam DA, Liu Y (2013). Reduced mitochondrial ROS, enhanced antioxidant defense, and distinct age-related changes in oxidative damage in muscles of long-lived *Peromyscus leucopus*. Am J Physiol Regul Integr Comp Physiol.

[15] Amaral S, Redmann K, Sanchez V, Mallidis C, Ramalho-Santos J, Schlatt S (2013). UVB irradiation as a tool to assess ROS-induced damage in human spermatozoa. Andrology.

[16] Feng Q, Li X, Peng J, Duan X, Fu Q, Hu Y (2012). Effect of gypenosides on DMN-induced liver fibrosis in rats. Zhongguo Zhong Yao Za Zhi.

[17] Kim J, Kim HY, Lee SM (2013). Protective effects of geniposide and genipin against hepatic ischemia/reperfusion injury in mice. Biomol Ther (Seoul).

[18] Sass G, Barikbin R, Tiegs G (2012). The multiple functions of heme oxygenase-1 in the liver. Z Gastroenterol.

[19] Zhong Z, Lemasters JJ (2004). Role of free radicals in failure of fatty liver grafts caused by ethanol. Alcohol.

[20] Dinnen RD, Mao Y, Qiu W (2013). Redirecting apoptosis to aponecrosis induces selective cytotoxicity to pancreatic cancer cells through increased ROS, decline in ATP levels, and VDAC. Mol Cancer Ther.

[21] Shamas-Din A, Kale J, Leber B, Andrews DW (2013). Mechanisms of action of Bcl-2 family proteins. Cold Spring Harb Perspect Biol.

[22] Renault TT, Teijido O, Antonsson B, Dejean LM, Manon S (2013). Regulation of Bax mitochondrial localization by Bcl-2 and Bcl-x(L): keep your friends close but your enemies closer. Int J Biochem Cell Biol.

[23] Renault TT, Manon S (2011). Bax: Addressed to kill. Biochimie.

[24] Zhou F, Yang Y, Xing D (2011). Bcl-2 and Bcl-xL play important roles in the crosstalk between autophagy and apoptosis. FEBS J.

[25] McIlwain DR, Berger T, Mak TW (2013). Caspase functions in cell death and disease. Cold Spring Harb Perspect Biol.

[26] Appelqvist H, Wäster P, Eriksson I, Rosdahl I, Ollinger K (2013). Lysosomal exocytosis and caspase-8 mediated apoptosis in UVA-irradiated keratinocytes. J Cell Sci.

[27] Qin Y, Vanden Hoek TL, Wojcik K (2004). Caspase-dependent cytochrome c release and cell death in chick cardiomyocytes after simulated ischemia-reperfusion. Am J Physiol Heart Circ Physiol.

[28] Kale J, Liu Q, Leber B, Andrews DW (2012). Shedding light on apoptosis at subcellular membranes. Cell.

[29] Zakeri Z, Lockshin RA (2008). Cell death: history and future. Adv Exp Med Biol.

[30] Bernardi P, Rasola A (2007). Calcium and cell death: the mitochondrial connection. Subcell Biochem.

